# Influence of the RelA Activity on *E. coli* Metabolism by Metabolite Profiling of Glucose-Limited Chemostat Cultures

**DOI:** 10.3390/metabo2040717

**Published:** 2012-10-12

**Authors:** Sónia Carneiro, Silas G. Villas-Bôas, Eugénio C. Ferreira, Isabel Rocha

**Affiliations:** 1 Institute for Biotechnology and Bioengineering (IBB), Centre of Biological Engineering, University of Minho, Campus de Gualtar, 4710-057 Braga, Portugal; Email: ecferreira@deb.uminho.pt (E.C.F.); irocha@deb.uminho.pt (I.R.); 2 Centre for Microbial Innovation, School of Biological Sciences, The University of Auckland, 3A Symonds Street, Auckland 1142, New Zealand; Email: s.villas-boas@auckland.ac.nz (S.G.V.B.)

**Keywords:** metabolomics, metabolite profiles, stringent response, relaxed phenotype

## Abstract

Metabolite profiling of *E. coli* W3110 and the isogenic Δ*rel*A mutant cells was used to characterize the RelA-dependent stringent control of metabolism under different growth conditions. Metabolic profiles were obtained by gas chromatography–mass spectrometry (GC-MS) analysis and revealed significant differences between *E. coli* strains grown at different conditions. Major differences between the two strains were assessed in the levels of amino acids and fatty acids and their precursor metabolites, especially when growing at the lower dilution rates, demonstrating differences in their metabolic behavior. Despite the fatty acid biosynthesis being the most affected due to the lack of the RelA activity, other metabolic pathways involving succinate, lactate and threonine were also affected. Overall, metabolite profiles indicate that under nutrient-limiting conditions the RelA-dependent stringent response may be elicited and promotes key changes in the *E. coli* metabolism.

## 1. Introduction

Bacteria are often used as microbial cell factories for delivering functional biomolecules with industrial or pharmaceutical interest. As most of these bioprocesses are metabolically demanding, it is critical to understand the physiological behavior of these organisms and to characterize their metabolic capabilities. Many studies have demonstrated that, under stressful conditions, their metabolic activities are not growth-related, which results in lower biomass yields and productivity [1,2]. Particularly, recombinant bioprocesses can be quite demanding for microbial cells due to the metabolic burden caused by the depletion of central metabolites like amino acids toward recombinant protein synthesis, which unbalances most central metabolic activities resulting in considerable productivity losses.

Under low nutrient conditions, cells usually engage in a multitude of cellular responses that allow their survival until growth resumes. Typically, the coordination of these cellular responses involves the global regulator guanosine-3',5'-bis-pyrophosphate (ppGpp), a core molecule that primarily triggers the stringent response [3,4,5,6]. Although the synthesis of ppGpp has been mainly associated with cellular responses to amino acid starvation, which in *E. coli* are mainly initiated by the activation of the ribosome-associated enzyme encoded by the *rel*A gene catalyzing the conversion of cellular GDP into ppGpp [7], recent studies have indicated that this molecule also accumulates during carbon starvation [8,9,10]. A second ppGpp synthetase, *i.e.*, the bifunctional enzyme SpoT that has both hydrolase and synthetase activities, has been described to be involved in ppGpp accumulation during carbon starvation [11,12], but its activity was shown to be much weaker than the one of the RelA enzyme [13]. This suggests that RelA may be central in the response to carbon starvation. It was thus suggested that these two nutritional stress phenomena are strictly correlated, the exhaustion of carbon often resulting in a rapid decrease in amino acids availability, entangling the activity of both enzymes [8]. Therefore, it is expected that RelA, directly or indirectly, interferes in the cellular responses to carbon-limited conditions. 

These phenomena have been implicated in recombinant bioprocesses using *E. coli* as an expression host [14]. It was found that ppGpp-deficient strains can maintain a metabolically productive state longer than the parent strains [15]. Thus, reducing the intracellular ppGpp levels seems to attenuate the pleiotropic effects on the metabolism, which is beneficial for the synthesis of foreign proteins. However, whether this is due to a less stress-responsive phenotype during recombinant production that eventually affects the metabolism, or to changes in the metabolic basis of this strain is still unclear. Despite the effects on the synthesis of foreign proteins, the impact of this regulator on the cellular metabolism of host strains needs to be characterized.

To investigate the metabolic state of *E. coli* cells and the role of the RelA enzyme (p)ppGpp synthetase in the *E. coli* responses to nutrient-limited growth conditions, a metabolomics approach was applied in this study. The intracellular metabolite profiles measured by gas chromatography–mass spectrometry (GC-MS) were used to assess the main metabolic changes resulting from different steady state growth conditions. Aerobic chemostat cultivations were performed at different dilution rates that provided for constant nutrient-limiting conditions specific for a single nutrient (*i.e.*, glucose) allowing steady state growth of cells (*i.e.*, at steady state the specific growth rate of cells is equal to the dilution rate). At these conditions, transient growth effects and other stress-induced responses are avoided that could mask effects resulting specifically from nutrient limitation. Three dilution rates were chosen based on previous results obtained in our laboratory that suggest that the effect of the nutrient limitation and, consequently, the RelA activity, is much lower at higher dilution rates. Thus, the steady state metabolism analyses of the wild-type and Δ*rel*A mutant cultures were performed at two low (0.05 and 0.1 h^−1^) and one higher (0.2 h^−1^) dilution rates. The aim of this study was to analyse the growth rate-dependent behaviour of *E. coli* cells and observe how the mutation in the *rel*A gene affects the cellular responses to nutrient-limiting conditions. This will provide us further information to evaluate ppGpp-deficient strains as potential hosts for recombinant *E. coli* bioprocesses. 

## 2. Experimental Section

### 2.1. Bacterial Strains and Growth Conditions

*E. coli* K12 W3110 (F-, *LAM-*, *IN*[*rrnD-rrnE*]*1*, *rph-1*) and the isogenic mutant Δ*rel*A (obtained from M. Cashel [13]) were grown under controlled conditions in a chemostat culture at 37 ºC, pH 7 and dissolved oxygen above 30%. The minimal medium consisted of 5 g·L^−1^ of glucose, 6 g·L^−1^ of Na_2_HPO_4_, 3 g·L^−1^ of KH_2_PO_4_, 0.5 g·L^−1^ of NaCl, 1 g·L^−1^ of NH_4_Cl, 0.015 g·L^−1^ of CaCl_2_, 0.12 g·L^−1^ of MgSO_4_•7H_2_O, 0.34 g·L^−1^ of thiamine, 2 mL·L^−1^ of trace-element solution (described elsewhere [16]) and 2 mL·L^−1^ of vitamins solution (described elsewhere [16]). The minimal medium was further supplemented with 20 mg·L^−1^ of L-isoleucine to grow the W3110 strain and 20 mg·L^−1^ of L-isoleucine and L-valine along with 25 mg·L^−1^ of kanamycin to grow the Δ*rel*A mutant strain.

Chemostat cultivations were carried out in a 3 L fermenter (BioFlo 3000, New Brunswick Scientific, USA) with a working volume of 1.5 L. The described minimal medium was continuously fed to the respective *E. coli* culture, at least for five residence times, at a given dilution rate (0.05, 0.1 and 0.2 h^−1^), and the working volume was kept constant by withdrawing the culture broth through level control. Steady-state conditions were verified by constant optical density and glucose measurements. The pH of the culture was maintained at 7.0 by adding 2.0 M NaOH and 2.0 M HCl. Dissolved oxygen was maintained above 30% saturation through a cascade mode controlling the agitation speed and airflow.

### 2.2. Analytical Techniques

The biomass concentration was determined by measuring culture absorbance (OD_600nm_) in a Jenway 6300 spectrophotometer and using a standard calibration curve (OD_600nm_ against cell dry weight (CDW)). In order to determine CDW, 10 mL of broth were filtered using 0.2 µm membrane filters and the filters with cell biomass were dried in the microwave to a constant weight [17]. For glucose and acetate analysis, culture broth was centrifuged at 8000 rpm for 15 min to remove the cell debris and the supernatant was collected. The glucose concentration in the culture broth was determined by the dinitrosalicylic acid (DNS) colorimetric method [18] and acetic acid was determined with an enzymatic test kit (R-Biopharm AG, Germany). 

#### 2.2.1. Quenching and Metabolite Extraction

For metabolomic analysis 3–4 sample replicates were used, following the sampling procedure described in [17]. In summary, 50 mL of fermentation broth samples were quickly harvested from the fermenter and immediately quenched in 200 mL of cold glycerol/saline solution (60%, v/v) at −23 °C. In order to extract intracellular metabolites, the recovered biomass was dissolved in methanol/water and then subjected to a series of freeze–thaw cycles. The supernatant was collected and kept at −80 ºC before lyophilization.

#### 2.2.2. Derivatization and GC-MS Analysis

The freeze-dried intracellular metabolite extracts were subjected to a chemical derivatization using methyl chloroformate (MCF) [19]. The derivatized samples were then analyzed in a GC7890 system coupled to a MSD 5975 detector (Agilent Technologies, Inc., Santa Clara, CA, USA). The GC was equipped with a ZB-1701 GC capillary column, 30m × 250mm id × 0.15 mm (film thickness) with a 5 m guard column (Phenomenex, Inc., Torrance, CA, USA) kept at 1.0 mL/min of helium. Further details of the analytical parameters can be found elsewhere [17].

### 2.3. Data Analysis

GC-MS results were analysed using AMDIS software [20]. Metabolites were identified using an in-house MS library [17]. The GC-peak intensities corresponding to each identified compound were normalized by both the GC-peak intensity of the internal standard (2,3,3,3-d4-alanine) and the biomass concentration (Table S1). The normalized peak intensities were then transformed into Z-scores, *i.e.,* standard scores that reflect how many standard deviations above or below the population mean a raw score is. Z-scores were calculated by subtracting the average peak intensity corresponding to a metabolite *K* among all the *n* samples (including replicates) in the set of experiments, from the peak intensity value (*I_K,i_*) for that metabolite in sample *i*, and dividing that result by the standard deviation of all measured peak intensities corresponding to that metabolite *K*, according to: 


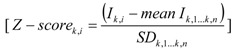
(1)

Further data processing and statistical analyses were performed with MATLAB (version 2009b, The Mathworks, Inc). The nonparametric two-way method, the Mack-Skillings test, was used to test the null hypothesis (*H_0_*) of no differences among experiments and to look for significant alterations between metabolic profiles that might be related to either factor: bacterial strain (Factor A) or dilution rate (Factor B). The design matrix for the Mack-Skillings test is provided in Table S2.

Metabolite profiles with p-values less than 0.01 were considered to have statistically significant differences between experimental conditions (for a 99% confidence level). We should be aware that there are slight differences in the media composition due to auxotrophic requirements (kanamycin and valine were added to the medium used to cultivate the mutant strain). Because we were concerned that the metabolic state of the two cultures would not be comparable if valine was also added to the wild-type culture (*i.e.*, while the mutant strain would use valine essentially to balance the inefficiency to naturally synthesize valine for biosynthetic purposes, in the wild-type, it would be used as a carbon source, which would interfere with the intracellular carbon distribution and, consequently, with the metabolic state of cells), we considered a stringent confidence level (99%) to reduce the influence of these environmental variations and metabolites in the biosynthetic pathway of valine which were not considered to be significant biomarkers.

Metabolite profiles that presented statistically significant changes regarding factor A (*i.e.*, bacterial strains) were further used to determine the degree of association between the metabolite profiles produced by the W3110 and Δ*rel*A *E. coli* cultures. We have applied a correlation analysis based on Pearson’s correlation coefficients (*r*) that measure the strength of the association between two conditions (e.g. bacterial strains), which can vary between 1 and −1 (*r* > 0 indicates a positive relationship, *r* = 0 indicates the absence of a relationship and *r* < 0 indicates a negative relationship). In this work, metabolite profiles with *r* < 0.6 were considered to correspond to a weak relationship between the metabolic behavior of W3110 and Δ*rel*A *E. coli* cultures.

Enrichment pathways analyses were performed using two bioinformatics tools: the Metabolite Biological Role (MBRole) a web-server tool for carrying out over-representation analysis of biological and chemical annotations in metabolomics data [21]; and the Pathway Activity Profiling (PAPi), an algorithm that measures metabolic pathway activities from metabolite profiles at different experimental conditions [22].

## 3. Results

### 3.1. Growth Parameters of E. coli Chemostat Cultures

Chemostat cultures of the *E. coli* W3110 and the isogenic Δ*rel*A mutant were run at different dilution rates (0.05, 0.1 and 0.2 h^−1^) and the determined growth parameters are shown in Table 1. 

**Table 1:** Growth parameters of the W3110 and Δ*rel*A mutant *E. coli* strains in aerobic glucose-limited chemostat cultures.

	W3110			Δ*rel*A mutant		
**Dilution rate (h^−1^)**	**0.05**	**0.10**	**0.20**	**0.05**	**0.10**	**0.20**
**Biomass yield (g_Biomass_.g_Glucose_^−1^)**	0.36±0.056	0.44±0.15	0.55±0.10	0.46±0.063	0.46±0.064	0.67±0.3
**Biomass (g_Biomass_.L^−1^)**	1.8±0.28	2.2±0.34	2.7±0.43	2.3±0.31	2.3±0.32	3.3±0.45
**Glucose (g_Glucose_.L^−1^)**	(1)	0.029±0.0086	0.040±0.0033	(1)	(1)	0.023±0.010
***q*** **_Glucose_** ** (g_Glucose_.g_Biomass_^−1^.h^−1^)**	0.14±0.021	0.23±0.076	0.36±0.063	0.11±0.015	0.22±0.030	0.30±0.13
**Acetate (g_Acetate_.L^−1^)**	(1)	(1)	0.34	(1)	(1)	0.02
***q*** **_Acetate_** ** (** **×** **10^3^)(g_Acetate_.g_Biomass_^−1^.h^−1^)**	-	-	25±3.8	-	-	1.1±0.15

(1) Undeterminable traces.

Overall, the biomass yields increased with the dilution rate, but the mutant strain exhibited a slightly higher biomass yield than the W3110 strain in the same conditions. In turn, the W3110 strain produced higher amounts of acetate than the mutant strain when growing at a higher dilution rate (0.2 h^−1^). Residual concentrations of glucose were also detected in the chemostat cultures, but only at higher dilution rates.

### 3.2. Metabolite Profiling

A chemical derivatization procedure was chosen in order to detect the main amino and non-amino organic acids and their precursors in the central carbon metabolism and fatty acid biosynthesis. The overall list of identified metabolites is presented in Table 2.

**Table 2:** List of the intracellular metabolites identified after GC-MS analysis.

*TCA intermediaries*	*Fatty acids*	*Amino acids*	*Others*
alpha-ketoglutarate (akg)	Hexanoate (hxa, n-C6:0)	Aspartate (asp)	Benzoate* (bnz)
cis-Aconitate (acon-C)	Octanoate (octa, n-C8:0)	Isoleucine (ile)	NADP(H)
Citrate (cit)	Decanoate (dca, n-C10:0)	Lysine (lys)	Nicotinate (nac)
Fumarate (fum)	Tetradecanoate (ttdca, n-C14:0)	Threonine (thr)	Phosphoenolpyruvate (pep)
Malate (mal)	10,13-Dimethyltetradecanoate (1013mlt)	Alanine (ala)	5-oxo-D-proline*(pyrglu)
Succinate (succ)	Pentadecanoate (pdca, n-C15:0)	Leucine (leu)	Malonate* (ma)
	14-Methylpentadecanoate (14mpdca)	Valine (val)	Itaconate* (itcon)
	Octadecanoate (ocdca, n-C18:0)	Glycine (gly)	Lactate (lac)
	Octadecenoate (ocdcea, n-C18:1)	Serine (ser)	
	9-*cis*,12-*cis*-Octadecadienoate (ocdcin, n-C18:2)	Glutamate (glu)	
		Proline (pro)	
		Phenylalanine (phe)	
		(*2S*)-2-isopropylmalate (3c3hmp)	
		N-Acetyl-L-glutamate (acglu)	

* Metabolites unknown to be synthesized by *E. coli*

**Figure 1:**
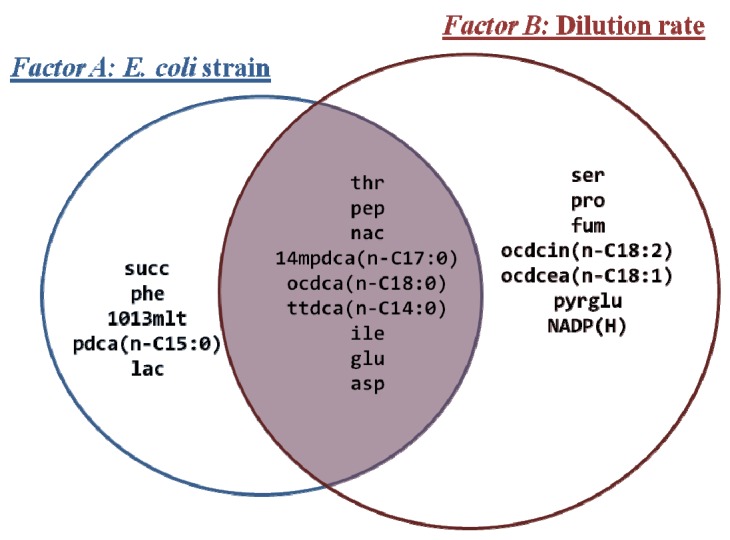
Venn diagram showing the list of the intracellular metabolites that were significantly changed (p-value < 0.01) according to either factors: A (*i.e.*, *E. coli* strain) or B (*i.e.*, dilution rate).

We have observed that both growth conditions (*i.e.*, dilution rates) and the genetic characteristics of *E. coli* strains (*i.e.*, presence/absence of the *rel*A gene deletion) induced significant alterations in the metabolite profiles of bacterial cultures, though the number of metabolites that had their levels significantly different depending on the dilution rate was slightly higher than when comparing *E. coli* strains (16 and 14 metabolites, respectively). Still, nine metabolites were commonly altered in both experimental conditions, indicating that metabolic states of cultures were profoundly affected in both cases. Almost 50% of the total metabolites were detected at significantly different levels in the mutant strain compared to the wild-type (Figure 1), meaning that, at the same steady state conditions, at least half of the detected metabolites presented significant differences in their abundances when comparing the two cultures. This suggests that enzymatic activities involving these metabolites are somehow influenced by this single mutation, leading to alterations in their levels. For instance, significant changes in amino and fatty acids levels, in particular tetradecanoate (*ttdca*, n-C14:0), pentadecanoate (*pdca*, n-C15:0), 10,13-dimethyltetradecanoate (*1013mlt*), octadecanoate (*ocdca*, n-C18:0), isoleucine (*ile*), threonine (*thr*), aspartate (*asp*) and glutamate (*glu*) were observed. Other metabolites that revealed interesting differences include N-acetyl-L-glutamate (*acglu*), lysine (*lys*), malate (*mal*), alpha-ketoglutarate (*akg*), itaconate (*itcon*) and malonate (*ma*); that were uniquely detected in the *E. coli* W3110 culture at a dilution rate of 0.1 h^−1^ (see Figure S1). Although these were not retrieved as statistically significant in the Mack-Skillings’s test, since they were not detected in any other samples, they may contribute to the differentiation between the metabolic behavior of W3110 and ∆*rel*A cultures. These metabolites are essentially associated with amino acid biosynthetic activities or metabolic regulation, like the itaconate (*itcon*) and malonate (*ma*), known to be enzymatic inhibitors of the isocitrate lyase, an enzyme associated with the glyoxylate cycle.

**Figure 2:**
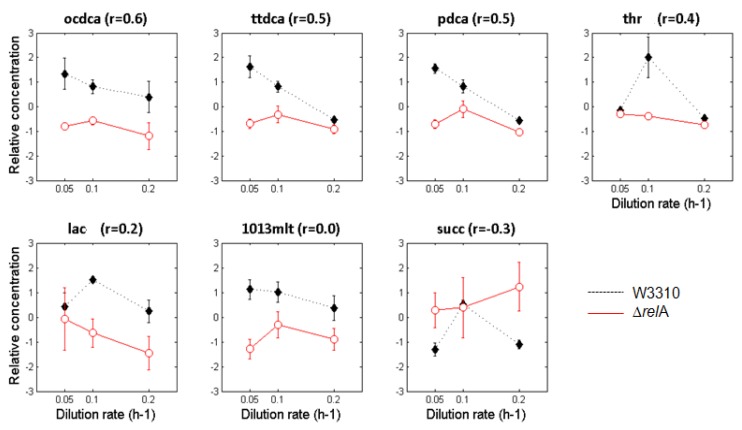
Metabolic patterns of the W3110 (represented by full diamonds and dashed lines) and Δ*rel*A (represented by open circles and red lines) *E. coli* cultures that presented low pairwise correlation coefficients (*r* < 0.6). The error bars shown in the line graphs represent the relative standard deviation among the 3–4 sample replicates. Only metabolites that presented significant changes according to the Mack-Skillings test for the strain factor (factor A) were considered in this analysis.

Besides differences in metabolite levels, we have paid attention to the changes in metabolite profiles produced by each *E. coli* strain at different dilution rates. We have evaluated this by estimating correlation coefficients based on the Pearson’s correlation, which identify uncorrelated patterns when comparing metabolite profiles along the three culturing conditions and between the two *E. coli* cultures. Figure 2 represents those metabolite profiles that showed significant uncorrelated patterns among cultures and the estimated pairwise Pearson’s correlation coefficients.

As illustrated in Figure 2, only one metabolite (succinate, *succ*) was found to have negatively correlated profiles, which means that the intracellular levels of this metabolite followed an opposite pattern in both *E. coli* strains. However, six other metabolites showed poorly correlated patterns that are essentially caused by discrepancies at lower dilution rates (*i.e.*, dilution rates of 0.1 and 0.05 h^−1^). Most of these uncorrelated profiles are associated with fatty acids, denoting that the coordination of fatty acids biosynthetic activities is somehow affected by the *rel*A gene mutation.

To understand how these specific metabolic alterations are related to changes in biochemical activities, metabolite profiles were translated into metabolic pathway activities. Two enrichment analyses were performed: the Metabolite Biological Role (MBRole) a web-server tool that uses biological and chemical annotations from different databases to highlight the biological role of metabolomics data; and Pathway Activity Profiling (PAPi), an algorithm that uses the metabolite profiles and KEGG database to compare the activities of metabolic pathways between different experimental conditions. While MBRole highlights metabolic activities that are over-represented in the metabolomics data, PAPi used the quantification of metabolite levels to determine pathways activity measured by the Activity Score (AS). In both analyses, pathways like “Aminoacyl-tRNA biosynthesis,” “ABC transporters,” “Citrate cycle (TCA cycle),” “Alanine, aspartate and glutamate metabolism” and “Fatty acid biosynthesis” were highlighted (see Tables S3 and S4). However, PAPi showed that, particularly at the dilution rate of 0.1 h^−1^, pathways such as “Aminoacyl-tRNA biosynthesis,” “ABC transporters,” “Nicotinate and nicotinamide metabolism,” “Sphingolipid metabolism” and “Sulfur metabolism” presented higher activity scores in the *E. coli* W3110 culture, whereas pathways of “Biosynthesis of unsaturated fatty acids” and “Alanine, aspartate and glutamate metabolism” showed lower activity scores. Clearly, metabolic pathway activities involving amino and fatty acids seem to be the most affected by the *rel*A gene mutation in these experiments.

**Figure 3:**
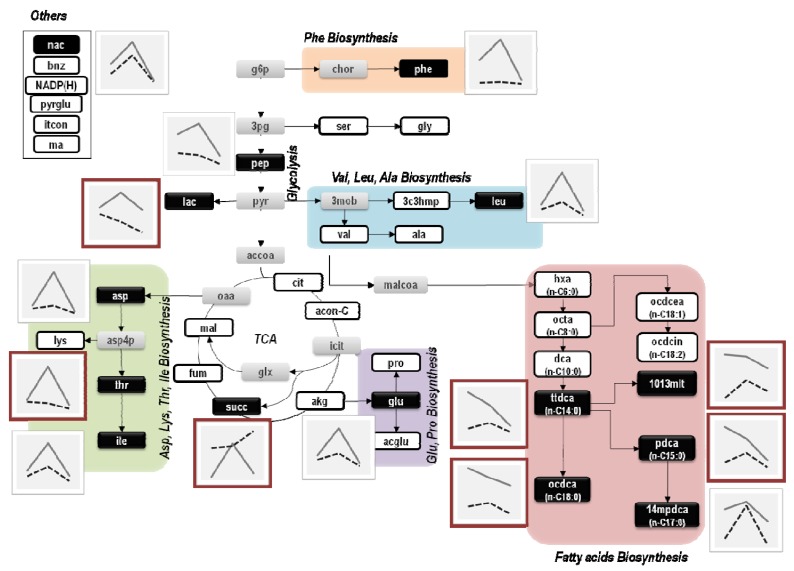
Representation of metabolic profiles on the metabolic map of *E. coli*. White and grey boxes represent metabolites that were respectively detected and undetected by the GC-MS analysis. Black boxes refer to metabolites that were found to change significantly according to the Mack-Skillings test. Each plot next to metabolite black boxes displays the corresponding metabolite profiles from each *E. coli* culture (dashed lines represent the metabolic profile of *E. coli* Δ*rel*A cultures and full lines represent the metabolic profile of *E. coli* W3110 cultures).

To illustrate these differences, metabolite profiles were also represented in the *E. coli* metabolic map that includes these major metabolic pathways (Figure 3).

As shown in Figure 3, alterations in metabolic profiles are essentially associated with amino and fatty acids biosynthetic pathways and, in most cases, are more evident at lower dilution rates. For instance, the profiles of octadecanoate (*ocdca*), tetradecanoate (*ttdca*), pentadecanoate (*pdca*) and 10,13-dimethyltetradecanoate (*1013mlt*) showed weak correlations when decreasing the dilution rate (from 0.1 to 0.05 h^−1^). Similarly, metabolites like succinate (*succ*), threonine (*thr*) and lactate (*lac*) showed opposite patterns compared to other metabolite profiles of *E. coli* Δ*rel*A mutant cultures. The succinate (*succ*) profile was the most divergent, showing clear differences between *E. coli* cultures at lower and higher dilutions rates.

## 4. Discussion

The growth rate-dependent regulation of the metabolism is fundamental to fine-tune the fueling and biosynthetic reactions in such a way that cells can rapidly adapt to the existing environmental conditions. Typically, the cellular metabolism increases with the growth rate to promote biomass formation in a more efficient way, as demonstrated by biomass yields in chemostat cultures (Table 1), *i.e.*, increased biomass yields were observed at higher dilution rates. However, it has been shown that at reduced dilution rates (e.g., 0.05 and 0.1 h^−1^), metabolism is not directly related to the growth rate, as cell growth becomes limited by cell-carbon supply [1]. As a result, the non-linearity observed in most metabolic profiles (Figure 2) must be an effect of the selected growth conditions that are inherently dependent on the energy-efficient use of the carbon substrate for biomass production. In this study, the majority of intracellular metabolite levels had a maximum at a dilution rate of 0.1 h^−1^, decreasing below and above this dilution rate. This was previously suggested to be associated with the extremely low residual glucose concentrations in glucose-limited cultures that triggers a series of cellular responses to adapt growth to these nutritional conditions [1,23].

According to Nanchen *et al.* [24], at a dilution rate of 0.1 h^−1^, large flux variations are verified in the metabolic network, in particular at the oxaloacetate node where two anaplerotic reactions converge. The carbon flux through the glyoxylate cycle (*i.e.*, an anaplerotic pathway that converts isocitrate to succinate or to malate via glyoxylate) is maximum at this dilution rate and decreases at higher dilution rates [1,25,26]. It was proposed [24,26,27] that at nutrient starvation conditions the cAMP-mediated catabolite repression of enzymes in the glyoxylate cycle is limited and the activity of the competing enzyme, *i.e.*, the isocitrate dehydrogenase, is decreased. As such, it is believed that anaplerotic reactions are stimulated in hungry *E. coli* cells and, at higher dilution rates, are restrained as a consequence of the increasing glucose concentrations and catabolite repression [28]. Clearly, depending on culture conditions, metabolic flux distributions can differ considerably, reflecting the variable efficiency of carbon utilization either for biomass formation or starvation responses.

Besides the aforementioned activities, a general increase in the central metabolism seems to occur at a dilution rate of 0.1 h^−1^, at which most metabolites reached its maximum levels. It is generally accepted that under steady state conditions an increase in metabolite levels would correspond to an increase in metabolic activities, since metabolism is fully balanced and no accumulation of intracellular metabolites is expected to occur due to a tight coupling of the anabolism and catabolism [29,30]. In this work, metabolic profiles of chemostat cultures of two *E. coli* strains (W3110 and the isogenic Δ*rel*A mutant) were determined by GC-MS analysis to explore the effects of different growth rate conditions on the *E. coli* metabolism, as well as to verify the involvement of RelA under such conditions. It has been proposed that under low growth, the RelA-dependent stringent control of many cellular activities is promoted, including some key metabolic activities [8,31,32,33,34,35]. Yet, little is known about the RelA-dependent ppGpp control over the *E. coli* metabolism and its influence on central metabolic activities. Our results show that metabolite pools were strongly affected by the *rel*A gene mutation as well as by the dilution rate. Though it was expected that metabolite levels would be altered with the dilution rate, due to the capacity of cells to alter their metabolism to cope with new growth conditions, the effect of the introduction of the single gene mutation (Δ*rel*A) was more difficult to predict. Differences observed in biomass yields have originally pointed to distinct metabolic behaviors between the two strains, *i.e.*, biomass yields were higher in the Δ*rel*A mutant cultures and were not linearly-dependent on the growth rate at lower dilution rates (0.05 and 0.1 h^−1^). Additionally, metabolomics analysis revealed that approximately 50% of the whole set of metabolites detected in this study presented significant changes between the *E. coli* W3110 and the Δ*rel*A mutant cultures (Figure 1). Most of these differences consisted in altered levels of amino acids and fatty acids indicating that the RelA-dependent ppGpp control of metabolic activities involving these metabolites might be affected. This seems to be the case of fatty acids like octadecanoate (*ocdca*), tetradecanoate (*ttdca*), pentadecanoate (*pdca*) and 10,13-dimethyltetradecanoate (*1013mlt*), that presented maximum levels at a dilution rate of 0.05 h^−1^ in the *E. coli* W3110 culture. Other examples include metabolites that were uniquely detected in the *E. coli* W3110 culture at a dilution rate of 0.1 h^−1^: *N*-acetyl-L-glutamate (*acglu*), lysine (*lys*), malate (*mal*), alpha-ketoglutarate (*akg*), itaconate (*itcon*) and malonate (*ma*) (see Figure S1). It seems that, at this particular dilution rate, the behaviour and regulation of metabolic activities associated (directly or indirectly) with those metabolites might be dependent on the activity of the RelA enzyme and, as a result, these metabolites could not be detected in the Δ*rel*A cultures, at least at traceable amounts. 

One of the most interesting phenotypic traits of the Δ*rel*A mutant strain is the reduced accumulation of acetate if compared to the control strain (0.02 and 0.34 g·L^−1^, respectively). Acetate was only detected in cultures at a dilution rate of 0.2 h^−1^, but differences between the two cultures reveal that the mutation influences the metabolic overflow metabolism. The overflow metabolism has an impact on biomass yields, as observed in our study, *i.e.,* the biomass yields of the mutant and wild-type cultures were 0.67 and 0.55 g of biomass per g of glucose, respectively, and may lead to growth arrest if the accumulation of by-products, such as acetate, reaches toxic levels. The acetate overflow metabolism has been recently investigated [36,37] and researchers found that acetate overflow results from the unbalanced synthesis and scavenging activities that are controlled by different mechanisms, including the CRP-cAMP-dependent catabolite repression. Under higher dilution rates (e.g., 0.2 h^−1^), the CRP-cAMP-dependent catabolite repression augments the overflow metabolism through the down-regulation of the acetyl−CoA synthetase that scavenges acetate. We hypothesize that this mutant is less responsive to this phenomenon and thus, acetate accumulation is reduced.

Besides these differences, it was found that some metabolite profiles correlate poorly when comparing *E. coli* W3110 and Δ*rel*A cultures at different dilution rates. This was mainly observed in fatty acids (octadecanoate (*ocdca*), tetradecanoate (*ttdca*), pentadecanoate (*pdca*) and 10,13-dimethyltetradecanoate (*1013mlt*)) that have also shown largest differences in the Mack-Skillings test for the strain factor (p-values of 0.0002) and threonine (*thr*), lactate (*lac*) and succinate (*succ*) profiles, which presented the lowest correlation coefficients (r < 0.6). This suggests that *E. coli* Δ*rel*A mutant cells are unable to maintain a close-to-wild-type behavior of the central carbon metabolism that may lead to important imbalances in metabolic functions.

It has been described that fatty acid biosynthetic genes are stringently controlled by ppGpp [38,39] and under nutrient-limiting conditions bacterial cells tend to adjust their cell wall composition [35,40,41]. Thus, the increasing levels of fatty acids at lower dilution rates are potentially associated with nutrient starvation responses, and in Δ*rel*A mutant cells, these cellular responses are evidently suppressed or simply not triggered. Interestingly, in the succinate (*succ*) profile, metabolite levels were higher in Δ*rel*A cultures, except at a dilution rate of 0.1 h^−1^. This suggests that in the Δ*rel*A culture, the metabolic activities involving this metabolite may be augmented, indicating a less stringent control of TCA enzyme activities or the activation of the glyoxylate cycle. However, the lack of information regarding other intermediaries of the TCA cycle does not support any further assumptions.

Overall, the stringent control of *E. coli* metabolism can be perturbed by the *rel*A mutation, in particular under slow growth steady states (0.05 and 0.1 h^−1^). Alterations in amino and fatty acids levels were significant, as was the poor correlation between several fatty acids profiles produced by the two *E. coli* cultures. In particular, fatty acids profiles were strongly divergent when decreasing the dilution rate (from 0.1 to 0.05 h^−1^), *i.e.*, while in the *E. coli* W3110 culture fatty acid levels increased, they decreased in the *E. coli* Δ*rel*A mutant culture. This supports the idea that the RelA enzyme is involved in the control of metabolic activities manipulating metabolite levels and thus, the metabolic state of cells. Many authors have stated that cells lacking the RelA-dependent stringent control have a relaxed phenotype [7,42,43,44], which is often characterized by a limitation of certain cellular processes, including central metabolic activities (e.g., fatty acids biosynthesis). Therefore, alterations observed in metabolite profiles might be explained by the lack of this enzyme and most likely a deregulation of certain metabolic functions.

Also, the effect of other regulators that play a role in the control of metabolism under nutrient-limited conditions cannot be disregarded. The CRP-cAMP transcriptional regulator is chiefly responsible for controlling metabolic fluxes under glucose limitation in *E. coli* cells [24]. This regulator responds to alterations in the intracellular cAMP levels resulting from glucose availability, which are higher at dilution rates below 0.1 h^−1^, and through the functional conversion of CRP into the active form CRP-cAMP that regulates the expression of various gene-encoding transporters and catabolic enzymes of sugars other than glucose [37]. At these conditions, we observed large differences between the Δ*rel*A mutant and wild-type cultures, which suggests that the single gene mutation influences the CRP-cAMP metabolic control. This phenomenon has been previously associated with the stringent response [8,45,46], indicating that ppGpp potentiates the expression of several stress response genes, namely the transcriptional regulator CRP that governs the catabolite repression. Thus, it was expected that Δ*rel*A mutants would be less effective in inducing anaplerotic reactions at a dilution rate of 0.1 h^−1^.

## 5. Conclusions

Metabolomics data have shown to be helpful in the interpretation of metabolic activities in many biological systems [15,47,48,49,50]. However, even with detailed knowledge about the overall metabolic reactions and their regulation, the interpretation of metabolic patterns is still not a trivial task. Analytical limitations in the detection of the whole set of metabolites within the cellular milieu are still a problem to fully characterize the metabolic state of a cell. For example, key metabolic nodes like isocitrate (*icit*), oxaloacetate (*oaa*) and glyoxylate (*glx*), would be important to evaluate the distribution of specific metabolic activities over the biochemical network.

Nevertheless, in this work, it was possible to address crucial metabolic alterations in response to different growth conditions, and more importantly, to verify that the RelA activity is fundamental in the coordination of several cellular processes, such as the biosynthesis of amino acids and fatty acids. These two metabolic activities were associated with the most remarkable differences between the two *E. coli* strains and exposed the range of metabolic deregulations that cells with relaxed phenotypes might exhibit. Yet, there is no evidence suggesting that the *rel*A mutation leads to impaired metabolic performances and is devoid of survival mechanisms. In fact, it was observed that biomass yields were higher in Δ*rel*A mutant cells. We believe that both the metabolic basis of these relaxed phenotypes and the inability to trigger several stress responses that would stall the cellular machinery [34,51], confer significant advantages to these strains as suitable hosts for recombinant production.

## Supplementary Files

Supplementary File 1 PDF-Document (PDF, 72 KB)
