# Supplementary material for: Influence of the RelA Activity on E. coli Metabolism by Metabolite Profiling of Glucose-Limited Chemostat Cultures
Source: Metabolites. 2012 Oct 12;2(4):717–32. doi: 10.3390/metabo2040717 (PMC3901239; doi:10.3390/metabo2040717)

Supplementary Material

Table S1 – Metabolomics dataset, which contains the abbreviation of the identified metabolites in the first column and their relative abundance in each sample of each condition in the subsequent columns.

Metabolites	W3110_D0.05-1	W3110_D0.05-2	W3110_D0.05-3	W3110_D0.05-4	W3110_D0.1-1	W3110_D0.1-2	W3110_D0.1-3	W3110_D0.2-1	W3110_D0.2-2	W3110_D0.2-3	W3110_D0.2-4	RelA_D0.05-1	RelA_D0.05-2	RelA_D0.05-3	RelA_D0.1-1	RelA_D0.1-2	RelA_D0.1-3	RelA_D0.2-1	RelA_D0.2-2	RelA_D0.2-3	RelA_D0.2-4
**NADP(H)**	0.08	0.09	0.06	0.10	0.36	0.41	0.46	0.10	0.08	0.08	0.11	0.15	0.13	0.15	0.23	0.21	0.18	0.07	0.09	0.09	0.10
**bnz**	0.45	0.35	0.27	0.37	0.34	0.28	0.41	0.42	0.62	0.41	0.48	0.91	0.38	0.90	0.40	0.33	0.36	0.59	0.34	0.60	0.22
**ala-L**	4.22	3.64	2.87	3.26	10.90	16.55	14.62	0.00	0.00	0.00	0.00	4.80	5.97	7.23	10.21	8.47	5.19	3.63	3.36	4.06	4.59
**gly**	1.16	1.42	1.25	1.34	2.28	2.51	2.09	1.80	1.83	1.52	1.37	0.69	1.08	1.10	1.50	1.08	1.14	1.84	0.00	1.91	1.49
**dca**	0.20	0.27	0.32	0.26	0.32	0.23	0.31	0.63	0.75	0.62	0.71	0.59	0.32	0.55	0.45	0.52	0.41	0.52	0.47	0.81	0.39
**3c3hmp**	0.18	0.17	0.18	0.20	0.46	0.77	0.67	0.00	0.00	0.00	0.00	0.00	0.00	0.00	0.27	0.22	0.22	0.00	0.00	0.00	0.00
**val-L**	3.77	3.13	2.41	3.17	12.34	20.22	17.56	2.57	2.78	2.10	1.93	6.04	5.35	6.18	14.21	11.10	10.01	1.74	1.56	2.29	1.90
**akg**	0.00	0.00	0.00	0.00	4.01	4.36	3.62	0.00	0.00	0.00	0.00	0.00	0.00	0.00	0.00	0.00	0.00	0.00	0.00	0.00	0.00
**leu-L**	2.55	2.29	2.34	2.41	4.95	5.17	4.61	2.26	1.93	1.77	1.66	1.97	1.78	2.24	3.21	2.55	1.98	1.35	1.41	2.09	1.23
**ile-L**	1.43	1.24	1.23	1.25	2.69	2.72	2.40	1.40	1.21	0.82	0.80	0.96	0.97	1.15	1.63	1.44	1.41	0.64	0.73	1.00	0.79
**pep**	0.65	0.64	0.43	0.51	1.07	0.66	0.52	0.10	0.22	0.16	0.13	0.11	0.16	0.32	0.25	0.13	0.12	0.00	0.00	0.00	0.00
**pro-L**	2.62	2.40	1.91	2.02	6.51	9.34	8.24	1.82	2.06	1.71	1.48	2.68	3.42	4.47	4.23	3.32	2.79	1.81	1.48	1.94	1.42
**acon-C**	0.28	0.47	0.30	0.36	2.08	2.86	2.05	0.14	0.31	0.19	0.12	0.09	0.11	0.14	0.15	0.12	0.10	0.20	0.14	0.18	0.20
**thr-L**	0.48	0.45	0.40	0.31	1.45	2.56	1.93	0.17	0.28	0.15	0.15	0.35	0.26	0.35	0.31	0.20	0.26	0.00	0.00	0.00	0.00
**ser-L**	0.05	0.05	0.09	0.06	0.63	0.85	0.69	0.08	0.07	0.07	0.07	0.14	0.12	0.14	0.21	0.17	0.17	0.00	0.00	0.00	0.00
**pyrglu**	5.49	5.68	3.74	4.48	13.55	22.39	19.35	1.93	3.06	2.47	1.65	2.33	4.71	6.82	13.45	7.73	7.55	1.08	0.74	1.20	0.79
**cit**	5.71	6.97	3.81	4.56	46.65	56.75	44.29	1.02	1.82	1.30	1.08	1.07	1.26	2.12	2.05	1.39	1.22	1.72	1.19	1.69	1.68
**asp-L**	24.25	24.17	15.79	19.49	76.57	115.84	92.02	8.04	9.26	6.55	5.69	5.02	15.24	22.08	27.17	18.25	16.03	2.89	2.64	3.62	2.95
**ttdca**	15.02	21.00	18.18	19.10	15.04	13.62	12.56	6.30	6.82	5.33	5.96	3.78	5.77	5.76	9.46	6.30	5.91	3.45	4.27	5.01	2.65
**glu-L**	22.45	22.21	17.01	18.69	51.86	74.65	63.19	7.58	10.91	6.95	5.49	4.81	15.59	18.95	28.64	19.15	22.49	3.76	2.31	2.92	3.11
**pdca**	8.86	10.42	9.55	9.54	8.07	7.05	6.27	2.66	2.59	1.91	2.62	1.32	2.30	2.23	5.31	3.49	3.19	0.93	0.40	1.41	0.82
**acglu**	0.00	0.00	0.00	0.00	0.31	0.31	0.25	0.00	0.00	0.00	0.00	0.00	0.00	0.00	0.00	0.00	0.00	0.00	0.00	0.00	0.00
**1013mlt**	38.87	50.12	47.21	46.58	49.98	40.81	42.42	39.68	43.15	28.91	34.71	11.19	19.09	19.46	35.82	25.60	24.09	21.03	23.80	26.32	13.81
**phe-L**	1.02	0.68	0.67	0.71	1.05	1.20	1.03	0.54	0.48	0.45	0.36	0.47	0.35	0.43	0.52	0.45	0.30	0.35	0.39	0.53	0.32
**14mpdca**	13.40	18.95	17.03	17.80	21.72	20.55	17.81	10.93	13.33	9.22	12.17	2.63	4.15	4.30	23.05	14.82	17.03	0.95	0.88	0.96	0.69
**ocdcea**	36.10	58.15	48.74	49.75	50.68	45.52	46.23	5.99	5.80	5.47	4.33	10.29	14.13	15.75	24.28	15.27	15.16	6.70	5.56	9.31	4.33
**ocdcin**	14.25	23.69	19.90	20.61	20.31	18.63	18.84	0.00	0.00	0.00	0.00	4.09	5.74	6.54	9.69	6.08	6.09	0.00	0.00	0.00	0.00
**ocdca**	2.80	4.21	3.86	3.57	3.43	2.94	3.07	2.71	3.62	2.54	2.24	1.68	1.78	1.77	2.03	1.75	2.02	1.48	1.32	2.00	0.76
**lys-L**	0.00	0.00	0.00	0.00	0.85	1.40	1.08	0.00	0.00	0.00	0.00	0.00	0.00	0.00	0.00	0.00	0.00	0.00	0.00	0.00	0.00
**ma**	0.00	0.00	0.00	0.00	0.80	1.01	0.98	0.00	0.00	0.00	0.00	0.00	0.00	0.00	0.00	0.00	0.00	0.00	0.00	0.00	0.00
**hxa**	2.31	2.18	2.15	2.61	2.90	1.52	2.30	2.70	3.87	2.91	3.03	9.30	5.36	8.23	3.34	2.93	2.57	3.37	2.70	5.06	2.39
**fum**	0.84	0.90	0.74	0.83	1.83	3.25	2.77	0.89	0.97	0.96	1.26	0.36	0.42	0.48	0.81	0.69	0.53	1.54	0.80	1.11	1.10
**succ**	1.97	1.69	1.23	1.49	3.62	3.46	3.20	1.68	2.05	1.56	1.87	4.09	2.38	3.07	4.16	4.13	1.56	4.48	3.16	3.16	5.69
**lac-L**	12.63	10.22	9.53	9.33	12.66	13.04	13.10	8.81	10.72	9.27	11.35	12.34	5.69	9.91	9.14	8.56	6.31	8.38	4.38	6.73	5.15
**mal-L**	0.00	0.00	0.00	0.00	1.24	2.26	1.76	0.00	0.00	0.00	0.00	0.35	0.34	0.44	0.00	0.00	0.00	0.00	0.00	0.00	0.00
**octa**	3.24	2.88	2.79	3.27	3.82	2.21	2.90	4.92	5.27	4.02	5.28	6.98	3.78	6.38	3.97	3.73	2.53	4.53	3.22	4.97	3.35
**itcon**	0.00	0.00	0.00	0.00	12.46	12.60	12.82	0.00	0.00	0.00	0.00	0.00	0.00	0.00	0.00	0.00	0.00	0.00	0.00	0.00	0.00
**nac**	1.59	1.78	1.63	1.86	3.12	2.98	2.35	0.96	0.94	0.69	1.00	1.43	0.95	1.24	2.75	2.11	1.59	0.73	0.86	0.93	0.72

Table S2 – Mack-Skillings test for two factor design. The design matrix captures the disproportionate number of replicates (either 3 or 4) in the group of experiments performed in this study.

		Factor B: **dilution rate**		
		0.05 h^{-1}	0.1 h^{-1}	0.2 h^{-1}
Factor A: **strain**	W3110	4	3	4
	Δ*rel*A	3	3	4

Table S3 – List of metabolic pathways retrieved from the Pathway Activity Profiling (PAPi) analysis and scores calculated for each pathway based on the metabolomics data.

Pathway	W3110			Δ*rel*A		
	**0.05 h^{-1}**	**0.1 h^{-1}**	**0.2 h^{-1}**	**0.05 h^{-1}**	**0.1 h^{-1}**	**0.2 h^{-1}**
**ABC transporters**	-16.86	188.67	-45.96	-28.38	3.02	-59.67
**Aminoacyl-tRNA biosynthesis**	-9.82	109.89	-26.77	-16.53	1.76	-34.75
**Nicotinate and nicotinamide metabolism**	-3.92	94.60	-23.41	-26.59	1.64	-24.92
**Sphingolipid metabolism**	-11.66	57.20	-10.64	-4.41	1.06	-18.09
**Sulfur metabolism**	-8.40	41.18	-7.66	-3.17	0.77	-13.02
**Novobiocin biosynthesis**	-16.36	79.83	-24.14	5.03	3.71	-25.92
**Taurine and hypotaurine metabolism**	-5.99	47.10	-16.13	-4.53	2.38	-11.59
**Cysteine and methionine metabolism**	-18.62	120.13	-40.27	-7.08	8.81	-32.50
**Glutathione metabolism**	-11.08	74.66	-14.36	-15.49	7.45	-24.53
**Valine, leucine and isoleucine biosynthesis**	-3.31	50.35	-13.41	-9.32	6.48	-18.92
**Pantothenate and CoA biosynthesis**	-9.47	57.42	-17.48	-5.76	11.75	-20.61
**Propanoate metabolism**	-14.28	43.88	-15.74	1.99	9.60	-11.58
**Valine, leucine and isoleucine degradation**	-9.48	84.00	-22.62	-13.70	18.44	-34.44
**D-Alanine metabolism**	-2.51	11.31	-7.10	0.78	3.34	-1.97
**Fatty acid metabolism**	34.26	55.31	-0.85	-51.17	44.06	-69.56
**Fatty acid elongation in mitochondria**	18.50	29.87	-0.46	-27.63	23.79	-37.56
**Glycolysis / Gluconeogenesis**	20.25	45.51	-3.84	-5.64	-15.18	-34.93
**Fatty acid biosynthesis**	28.72	22.08	8.02	-19.52	-7.30	-33.18
**Phenylalanine metabolism**	-18.74	60.77	-19.75	3.63	-20.09	5.26
**Phenylalanine, tyrosine and tryptophan biosynthesis**	21.61	46.84	-13.10	-13.07	-14.13	-23.24
**Phosphotransferase system (PTS)**	42.09	73.26	-22.65	-15.32	-20.83	-47.26
**Phosphonate and phosphinate metabolism**	41.17	71.66	-22.16	-14.99	-20.38	-46.24
**Purine metabolism**	-25.69	140.29	30.07	-81.42	-34.13	-22.92
**Thiamine metabolism**	-7.26	39.65	8.50	-23.01	-9.65	-6.48
**Lysine degradation**	-15.71	90.70	-1.46	-29.94	-17.86	-15.00
**Citrate cycle (TCA cycle)**	-5.04	38.53	-9.08	-6.33	-7.06	-4.73
**Biotin metabolism**	-4.28	25.68	-4.28	-4.28	-4.28	-4.28
**Pyrimidine metabolism**	-23.36	140.19	-23.36	-23.36	-23.36	-23.36
**Pentose and glucuronate interconversions**	-21.84	131.04	-21.84	-21.84	-21.84	-21.84
**Vitamin B6 metabolism**	-12.71	76.24	-12.71	-12.71	-12.71	-12.71
**Ascorbate and aldarate metabolism**	-18.66	111.98	-18.66	-18.66	-18.66	-18.66

**Methane metabolism**	-6.11	153.29	-21.32	-25.59	-25.12	-49.50
**Pyruvate metabolism**	7.52	58.34	-11.40	-7.61	-8.95	-27.45
**Glyoxylate and dicarboxylate metabolism**	-23.05	98.09	-25.81	-9.49	-14.38	-6.81
**Two-component system**	-13.66	58.54	-15.13	-8.17	-7.81	-3.13
**Lysine biosynthesis**	-9.60	74.46	-14.24	-11.93	-9.75	-15.74
**C5-Branched dibasic acid metabolism**	-7.93	74.08	-14.51	-13.17	-8.94	-16.54
**beta-Alanine metabolism**	-7.90	71.91	-14.65	-11.29	-8.12	-16.82
**Glycine, serine and threonine metabolism**	-11.80	101.96	-13.27	-19.97	-9.67	-29.16
**Porphyrin and chlorophyll metabolism**	-15.94	245.05	-30.12	-60.85	-22.30	-75.37
**Biosynthesis of secondary metabolites**	-220.94	2334.56	-488.19	-318.57	-205.79	-648.52
**Oxidative phosphorylation**	-11.16	20.47	-7.70	-5.25	-1.76	8.77
**Tyrosine metabolism**	-52.99	97.22	-36.59	-24.96	-8.34	41.64
**Arginine and proline metabolism**	-23.94	198.72	-41.82	-33.77	-15.09	-46.63
**Cyanoamino acid metabolism**	-12.32	87.09	-9.29	-19.98	-6.54	-23.82
**Butanoate metabolism**	-17.94	70.97	-19.80	-14.05	-4.52	-1.57
**Bacterial chemotaxis**	-0.68	13.58	-3.29	-2.00	-0.77	-4.14
**Nitrogen metabolism**	-3.42	51.52	-7.30	-13.59	-2.89	-15.55
**Histidine metabolism**	-7.52	99.76	-22.97	-15.85	-5.28	-28.48
**D-Glutamine and D-glutamate metabolism**	-2.39	27.23	-6.10	-4.49	-1.39	-7.52
**Biosynthesis of unsaturated fatty acids**	61.51	59.44	-20.63	-35.20	-0.51	-58.68
**Alanine, aspartate and glutamate metabolism**	-9.30	44.98	-14.85	-6.43	-0.09	-4.70

Activity scores (AS) are calculated for each pathway based on the number of metabolites identified from each pathway and their relative abundances. As a result, the activity score represents the likelihood of a metabolic pathway to be active, meaning that the higher the score the lower the activity. Negative activity scores are due to the use of z-scores instead of positive metabolite abundances, which means that the original relative abundance was below the mean.

Table S4 – List of metabolic pathways retrieved from the MBRole analysis and p-values calculated for each pathway based on the metabolomics data.

MBRole computes the statistical significance of each annotation found in the set of compounds, using the background set. This is provided as a p-value (column 2 - *p-val*), or the probability of obtaining such a number of compounds or more with a particular annotation if we take a random set of the same size from the background set. This p-value is also adjusted for multiple testing using the false discovery rate (FDR) method by Benjamini and Hochberg (1995) (column 3 - *adjusted p-val*). Finally, MBRole uses a background set that is a reference set (column 4 - *in bckgnd*) for assessing the significance of a given annotation found in your compounds of interest (column 5 - *in set*). A given annotation could have a high frequency (column 6 - *%*) in your set of compounds simply because it is frequent in the whole metabolome. This is why a reference set is needed for assessing significance.

**Pathway**	**p-val**	**adjusted p-val**	**in bckgnd**	**in set**	**%**	**Compounds**[¥]
**Aminoacyl-tRNA biosynthesis**	3.3E-12	1.9E-10	75	12	34.3	C00148 C00079 C00407 C00041 C00183 C00025 C00065 C00037 C00123 C00047 C00188 C00049
**ABC transporters**	2.3E-11	6.6E-10	90	12	34.3	C00041 C00047 C00079 C00049 C00123 C00183 C00407 C00037 C00025 C00065 C00188 C00148
**Citrate cycle (TCA cycle)**	3.4E-10	6.7E-09	20	7	20	C00417 C00074 C00158 C00122 C00042 C00149 C00026

**Alanine, aspartate and glutamate metabolism**	7.0E-08	1.0E-06	24	6	17.1	C00049 C00026 C00122 C00042 C00041 C00025
**Valine, leucine and isoleucine biosynthesis**	5.8E-06	5.8E-05	28	5	14.3	C02504 C00123 C00407 C00188 C00183
**Fatty acid biosynthesis**	6.1E-06	5.8E-05	49	6	17.1	C00249 C00712 C06423 C01571 C06424 C01530
**Two-component system**	7.0E-06	5.8E-05	29	5	14.3	C00049 C00122 C00149 C00158 C00042
**Biosynthesis of secondary metabolites**	2.7E-05	1.8E-04	1023	22	62.9	C00047 C00186 C00149 C02504 C00123 C00042 C00148 C00065 C00158 C00079 C00025 C00074 C00037 C00624 C00180 C00183 C00026 C00122 C00188 C00417 C00049 C00407
**Glyoxylate and dicarboxylate metabolism**	5.7E-05	3.3E-04	44	5	14.3	C00149 C00026 C00158 C00417 C00042
Pyruvate metabolism	2.3E-04	1.1E-03	32	4	11.4	C02504 C00074 C00149 C00186
**C5-Branched dibasic acid metabolism**	2.3E-04	1.1E-03	32	4	11.4	C00026 C00490 C00417 C00025
**Glutathione metabolism**	4.6E-04	2.1E-03	38	4	11.4	C00037 C00005 C00025 C01879
**Butanoate metabolism**	5.6E-04	2.3E-03	40	4	11.4	C00122 C00042 C00026 C00025
**Phenylalanine metabolism**	9.7E-04	3.7E-03	46	4	11.4	C00079 C00180 C00122 C00042
**Arginine and proline metabolism**	1.1E-03	4.0E-03	82	5	14.3	C00025 C00148 C00122 C00049 C00624
**Glycine, serine and threonine metabolism**	1.2E-03	4.2E-03	49	4	11.4	C00065 C00188 C00049 C00037
**Biosynthesis of unsaturated fatty acids**	1.8E-03	5.7E-03	54	4	11.4	C00249 C01530 C00712 C01595
**Nitrogen metabolism**	2.0E-03	6.0E-03	26	3	8.6	C00049 C00025 C00037
**Lysine biosynthesis**	3.6E-03	1.0E-02	32	3	8.6	C00026 C00047 C00049
**Propanoate metabolism**	5.0E-03	1.4E-02	36	3	8.6	C00183 C00042 C00186
**D-Glutamine and D-glutamate metabolism**	5.9E-03	1.5E-02	12	2	5.7	C00026 C00025
**Cyanoamino acid metabolism**	7.3E-03	1.8E-02	41	3	8.6	C00049 C00065 C00037
**Valine, leucine and isoleucine degradation**	7.3E-03	1.8E-02	41	3	8.6	C00123 C00407 C00183
**Nicotinate and nicotinamide metabolism**	8.8E-03	2.0E-02	44	3	8.6	C00049 C00153 C00122
**Histidine metabolism**	8.8E-03	2.0E-02	44	3	8.6	C00026 C00049 C00025
**Oxidative phosphorylation**	1.0E-02	2.2E-02	16	2	5.7	C00122 C00042
**Cysteine and methionine metabolism**	1.7E-02	3.5E-02	56	3	8.6	C00065 C00041 C00049
**Benzoate degradation via CoA ligation**	1.8E-02	3.6E-02	57	3	8.6	C00122 C00180 C00042
**Pantothenate and CoA biosynthesis**	2.8E-02	5.2E-02	27	2	5.7	C00183 C00049
**Phenylalanine, tyrosine and tryptophan biosynthesis**	2.8E-02	5.2E-02	27	2	5.7	C00074 C00079

**Styrene degradation**	2.8E-02	5.2E-02	27	2	5.7	C00186 C00122
**Glycolysis / Gluconeogenesis**	3.7E-02	6.3E-02	31	2	5.7	C00074 C00186
**beta-Alanine metabolism**	3.7E-02	6.3E-02	31	2	5.7	C00049 C00383
**Methane metabolism**	4.4E-02	7.2E-02	34	2	5.7	C00065 C00037
**Lysine degradation**	7.8E-02	1.2E-01	47	2	5.7	C00047 C00037
**Porphyrin and chlorophyll metabolism**	1.3E-01	2.0E-01	126	3	8.6	C00037 C00188 C00025
**Tyrosine metabolism**	1.7E-01	2.6E-01	76	2	5.7	C00042 C00122
**Fatty acid metabolism**	1.0E+00	1.0E+00	50	1	2.9	C00249
**Bacterial chemotaxis**	1.0E+00	1.0E+00	6	1	2.9	C00049
**Novobiocin biosynthesis**	1.0E+00	1.0E+00	37	1	2.9	C00148
**Pyrimidine metabolism**	1.0E+00	1.0E+00	59	1	2.9	C00383
**Benzoate degradation via hydroxylation**	1.0E+00	1.0E+00	48	1	2.9	C00180
**Phosphonate and phosphinate metabolism**	1.0E+00	1.0E+00	45	1	2.9	C00074
**Biotin metabolism**	1.0E+00	1.0E+00	11	1	2.9	C00047
**Purine metabolism**	1.0E+00	1.0E+00	92	1	2.9	C00037
**Biphenyl degradation**	1.0E+00	1.0E+00	23	1	2.9	C00180
**Sulfur metabolism**	1.0E+00	1.0E+00	18	1	2.9	C00065
**Vitamin B6 metabolism**	1.0E+00	1.0E+00	32	1	2.9	C00026
**Selenoamino acid metabolism**	1.0E+00	1.0E+00	30	1	2.9	C00041
**Taurine and hypotaurine metabolism**	1.0E+00	1.0E+00	20	1	2.9	C00041
**D-Alanine metabolism**	1.0E+00	1.0E+00	6	1	2.9	C00041
**Toluene and xylene degradation**	1.0E+00	1.0E+00	48	1	2.9	C00180
**Sphingolipid metabolism**	1.0E+00	1.0E+00	25	1	2.9	C00065
**Ascorbate and aldarate metabolism**	1.0E+00	1.0E+00	47	1	2.9	C00026
**Thiamine metabolism**	1.0E+00	1.0E+00	26	1	2.9	C00037
**gamma-Hexachlorocyclohexane degradation**	1.0E+00	1.0E+00	41	1	2.9	C00042
**Phosphotransferase system (PTS)**	1.0E+00	1.0E+00	46	1	2.9	C00074

¥ KEGG IDs

Figure S1- Relative concentrations of metabolites that were undetected in chemostat cultures, except in the *E. coli* W3110 grown at a dilution rate of 0.1 h^{-1}.

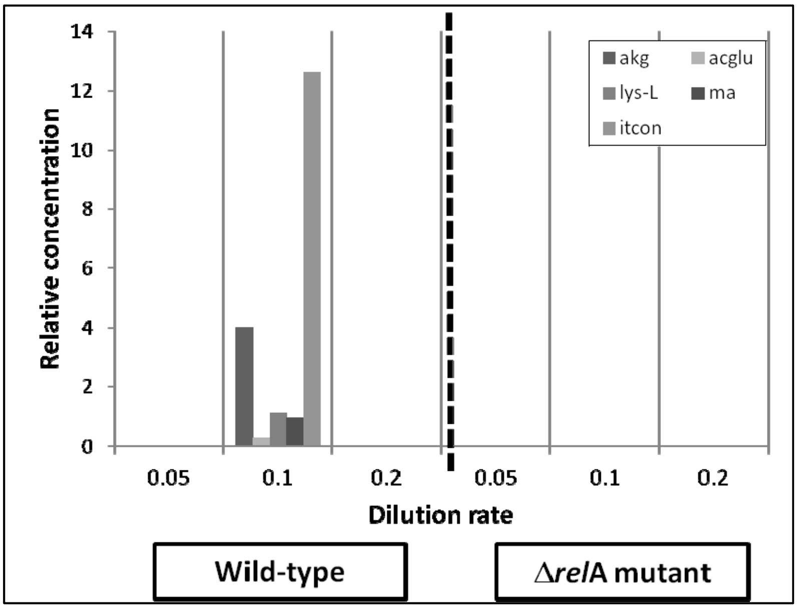

Supplement: Supplementary File 1 — PDF-Document (PDF, 72 KB) [file metabolites-02-00717-s001.pdf]
